# Global Psychological Implications of Severe Acute Respiratory Syndrome Coronavirus 2 (SARS-CoV-2) and Coronavirus Disease-2019 (COVID-19). What Can Be Learned From Italy. Reflections, Perspectives, Opportunities

**DOI:** 10.3389/fpsyg.2020.01836

**Published:** 2020-07-24

**Authors:** Andrea De Giorgio

**Affiliations:** Faculty of Psychology, eCampus University, Novedrate, Italy

**Keywords:** pandemic, psychosocial factors, stress, quarantine, black swan

## Abstract

On December 31, 2019, the Chinese authorities announced that in the city of Wuhan, Hubei Province, central-eastern China, a cluster of pneumonia cases of unknown etiology had developed. A new coronavirus (SARS-CoV-2) that causes serious problems like pneumonia and even death, has been discovered. This new disease (COVID-19) has spread also in Italy starting from the first recognized case on February 20. Beyond its biological implications, this coronavirus allows us many psychological reflections. A new virus is indeed a potentially serious problem for mankind, but it can also be an opportunity to bring the focus back to us, to observe what is happening, who we are and how we are reacting both as individuals and as a population. Even positive implication of this pandemic was discussed.

“It is important not to underestimate the small opponents:
you can see an elephant, a little mosquito, but not a virus”

The novel coronavirus disease (COVID-19) – first revealed in late December 2019 in the city of Wuhan of Hubei Province (Wang et al., 2020a) – has recently been considered pandemic by World Health Organization (World Health Organization [WHO], 2020).

At the moment if you Google “COVID-19” (i.e., the disease), the search engine returns about 5.09 billion results, and about 3 billion if you Google “Coronavirus,” a term used to describe a large family of viruses known to cause several respiratory syndrome (e.g., SARS; MERS). A search for “SARS-CoV-2” (i.e., name of the virus) gives fewer results, just under 357 million, but this is easily explained by the fact that the general population tends to look for the terms most used by the media: “Coronavirus” and “COVID-19.” Inspired by an Editorial appeared in The New England Journal of Medicine (Jones, 2020) an idea was born, that is to compare on Google the terms related to novel coronavirus and another very known virus and its related syndrome: “HIV” and “AIDS,” respectively. The numbers are impressive: “AIDS” gives just under300 million results and “HIV” just over 231 million.

That said, it is possible to make a biological–psychological comparison, taking into account the rapidity of the effects: faster in the case of SARS-CoV-2 and slower in the case of HIV. In fact, a virus is biologically all the stronger the more it is able to remain latent in the human body, and this is the case with HIV, because in doing so it is more “silently contagious.” Conversely, SARS-CoV-2 is a “noisily infectious” virus, which makes it easier to trace, making restrictive measures all the more urgent. Without entering into discussions of an epidemiological nature which, in any case, are not widely understood by the general public (e.g., the difference between mortality and deadliness) what psychological considerations can be made?

In Italy, the arrival of the virus has unleashed an unprecedented media bombardment and thrown our authorities in confusion. In an initial period lasting about 10 days there was excessive media exposure on the part of the Prime Minister, whose continuous updates on the spread of the COVID-19 triggered alarmism followed by mass behaviors such as long queues outside supermarkets to raid all kinds of product (including toilet paper) and fear of entering Chinese-run businesses or of frequenting ethnic Chinese people, even if born in Italy. Moreover, due to the media bombardment, there are at least three problems: (i) much useful information for the general population is hidden; (ii) the load of information about COVID-19 leads population to be more confused (e.g., virologists, immunologist, and epidemiologists on TV are giving conflicting information on the use of masks or gloves); and (iii) authorities and associated health experts in their public appearances have often used catastrophing and emphasizing style of communication for some situations associated with the pandemic COVID-19.

It is possible to think that this information approach was necessary and urgent in order to change social patterns of behavior (i.e., social distancing; use protective measures; and general reduction of citizen transfers). This load of information, although sometimes confused, may have been helpful to induce worry in the general population so that social patterns of behavior changed. The other side of the coin is that because of this pattern of information, general population could find an answer to worry and justify a given behavior (e.g., do not use the mask because it is harmful; Allington et al., 2020; Bao et al., 2020; Liu, 2020).

The extreme difficulty with which our brain processes excess and complex information contributes to unjustified worry and alarmism (Feng et al., 2015). Because we struggle to access and properly analyze a media bombardment of this kind, we tend to create artificial logical structures that include only the information that enables us to develop representative models of reality.

This can lead, for example, to defense mechanisms in social relations, leading us to associate a terrorist attack with a man speaking Arabic, or to think someone is affected by COVID-19 just because they are Chinese or because they have sneezed. This causes phenomena such as discrimination and the construction of stereotypes. In addition to this, during a situation of media uncertainty (i.e., retractions and continuous updates) such as that brought about by this coronavirus, a lot of information is ignored or mistaken for fake news (Shimizu, 2020).

Thus the huge media bombardment and the vast quantity of results from googling “Coronavirus,” “COVID-19,” and “SARS-CoV-2” give the impression of a psychological and emotional contagion (Kramer et al., 2014; Ferrara and Yang, 2015) so that in literature appears a new term a neologism “Coronaphobia” (Asmundson and Taylor, 2020). This emotional involvement that is capable of generating distress, altered risk perception, and also leading to cyberchondria, a clinical phenomenon characterized by repeated Internet searches for medical information which leads to excessive concerns about physical health (Mathes et al., 2018). This phenomenon may explain the vast quantity of Google search results on a given disease, which also depends on the fear of contracting or avoiding it, as in the case of COVID-19.

Cyberchondria is positively associated with symptoms of anxiety (Mathes et al., 2018) and may lead to increased levels of distress, worry, unnecessary medical expenses (Fergus, 2014), and altered risk perception (Rübsamen et al., 2015). Wang et al. (2020b) investigated psychological indexes in Chinese people following COVID-19 outbreak demonstrating a psychological impact from moderate-to-severe; in particular one-third of them reported moderate-to-severe anxiety.

However, even before SARS-CoV-2 made the “species jump,” anxiety disorders were one of the most common classes of disorders worldwide and the sixth leading contributor to disability worldwide (Baxter et al., 2014). In America it is estimated that adult people with anxiety disorder is about 40 million, with lifetime morbid risk estimated at 41.7% in the general population (Kessler et al., 2012). These disorders significantly impact quality of life and functioning across life domains (Norberg et al., 2008). And add to this the anxiety disorders are associated with psychiatric and physical comorbidity (e.g., Kuvačić et al., 2018), increases in medical service utilization, and significant societal costs associated with loss of productivity and work impairment (e.g., Barattucci et al., 2019).

In addition, attribute-framing bias can be added to cyberchondria (Kreiner and Gamliel, 2019). This bias leads us to evaluate positively framed objects more favorably than the same objects framed negatively. For example, it is the dynamic that leads us to choose a yogurt that promises us 20% fresh fruit, ignoring the concentrate that constitutes an abundant 50% of the product. It is not a major problem as long as it only concerns our breakfast, but it is much more serious when, during an epidemic, 2% of deaths leads us to ignore the 98% that survived.

The brain structures that mediate fear-related emotions, such as anxiety, are very complex and involve archaic areas of the brain such as the amygdala, hippocampus, ventromedial hypothalamus, insular cortex, etc (for review, Garcia, 2017), structures that are activated specifically but not exclusively to saving our lives and that make us feel anxious precisely in order to protect us from a potential danger, even if in the case of anxiety the danger is future and not present. After all, the literature shows our natural predisposition to remember unpleasant events and negative information, activating the brain areas that underlie them in such a way as to anticipate the danger (e.g., Kellermann, 1984).

Specifically, the amygdala and its neural network mediate emotional learning and behavior, playing a major role in mediating fear and other emotions linked to anxiety disorders such as generalized anxiety disorder, panic, substance or medication-induced anxiety, social anxiety disorder, and others. These emotions and their neural network are controlled by frontal areas of the brain that are able to deactivate or reduce the activity of the areas related to emotional activation (e.g., Guendelman et al., 2017). Moreover, when human beings are anxious their perception of reality, and therefore of the disease, can vary, and it has been demonstrated that anxiety is associated with difficulties in decision making (for review, Bishop and Gagne, 2018; Zhang and Gu, 2018), but it has also been demonstrated that emotional regulation is followed by less risky decisions (Morawetz et al., 2019).

The numbers we have described on Google searches clearly show us how high the SARS-CoV-2 anxiety is at this particular moment. This, of course, should not necessarily be seen as a problem, since it is not unusual to feel temporary anxiety when facing stressful situations, uncertainty, or extreme challenges. The emotions of anxiety and fear in confronting a real threat are part of the survival instinct. Anxiety can make us be more careful about taking a number of precautions that prevent SARS-CoV-2 infection, but the question is: why does this not happen, or no longer happen, for HIV? It is possible to think that the problem is both mediatic and related to the perception of the looming new danger.

Taleb (1960) described the so-called “black swan” effect (2008), i.e., the strong impact that some rare and unpredictable events have on the mind and the tendency of people to retrospectively find simplistic explanations for these events. An example of this effect can be given by September 11, a date before which no one would have ever expected anyone to fly a plane into a building in order to carry out a terrorist attack. The black swan effect seems to be paradoxically fitting for SARS-CoV-2 despite the fact that history tells us that this event is not a real “black swan,” because a new virus is certainly neither rare nor unpredictable. Think for example, about Ebola (see for review, Jacob et al., 2020), SARS-CoV (Sun et al., 2020), H1N1, H5N1, and H3N2 (Guarner and Falcón-Escobedo, 2009), Hendra and Nipah (Eaton et al., 2006), etc.

However, fear and anxiety linked to death are resurgent globally every time a new virus appears in the world and becomes pandemic, paradoxically becoming first a “black swan,” and then decreasing and leading to a sort of “psychological habituation” (Ziferstein, 1967). This can explain why HIV is so “psychologically silent” in Google searches, because there is a perception that the virus has been defeated (in truth it has only become a chronic condition) since, thanks to treatment (and its accessibility), the life expectancy of HIV+ has increased in the world, even though people still die of AIDS, especially in sub-Saharan Africa, and often from opportunistic diseases (UNAIDS, 2019).

Yet such a strong reaction to a virus has not been seen before in Italy. Here, the situation is more complicated than we thought: in the Northern Italy, Lombardia region, two large clusters of outbreaks have spread starting from a 38-year-old man from the city of Codogno, who presented at the hospital on February 20. The virus is spreading very quickly and efficiently so that many regions are increasing intensive care beds, revolutionizing entire hospital wards. Our healthcare professionals are facing disease pulling 12-h shifts in critical situations and this phenomenon is leading to serious psychological distress in this population here (Anmella et al., 2020; Barello et al., 2020; De Giorgio, 2020; ISS, 2020; Ramaci et al., 2020a) as well as in other countries (Bohlken et al., 2020; Heath et al., 2020; Ornell et al., 2020; Tsamakis et al., 2020).

Italy’s government measures are very severe and extraordinary and the country is in lockdown since two months (De Giorgio, 2020).

In the other nations a similar framework is showing up and as already written by Crawford et al. (2016): “*The world remains ill prepared to handle sustained responses and global pandemics*,” and this also seems to apply psychologically: a previous virus does not make us immune from the fear, distress and anxiety that causes the next one. For this reason it is right, as is happening in our country, to apply the correct prophylactic measures (i.e., “quarantine”) in order to dilute the spread of the pathogen, even if poorly tolerated by the population (Brooks et al., 2020).

However, there is yet another opportunity to change our psychological approach to events of this kind. First of all, there should be more attention to research funding, which is drastically scarce in Italy, and to public health, which is a source of absolute pride in our country: anyone who falls ill in Italy, wherever they come from, even if they do not have an identity document or a credit card, is treated for free.

Secondly, this umpteenth “black swan” brought about by a virus once again makes us aware of the importance of education in emotional regulation. Knowing how to manage emotions well, for example, through mindfulness practices – which can increase well-being and decrease anxiety and depression in healthy, professionals and patient populations (De Giorgio et al., 2017a, b; Grazzi et al., 2017; Padovan et al., 2018; Ramaci et al., 2020b) – can allow people to have a balanced reaction and a clearer understanding of the phenomenon, thanks also to the neuro-bio-physiological effects that these practices have on the brain. In fact, it has been widely proven in the literature that these types of practices are able to structurally and functionally modify the areas of the brain that regulate the networks related to emotions (see for review, Young et al., 2019) and even reduce the size of the amygdala (Taren et al., 2013).

It is also necessary to practice “positive emotional contagion.” In fact, it has been widely demonstrated (see, e.g., Cirelli et al., 2018) that distress is closely related to anxiety, and this is also confirmed from the neurobiological point of view (Daviu et al., 2019). At this current time the media talk about nothing but infections and deaths, and this can help to feed the vicious cycle of anxiety-distress. The effect that distress has on the immune system must be taken into great consideration. Indeed, the effects of distress on diseases such as viral or bacterial infection are often associated with several immune dysregulation (see, e.g., Powell et al., 2013). Moreover, the protective role of dispositional optimism has also been demonstrated (Levy et al., 2019), and has been linked to lower levels of inflammation markers, better antioxidant levels and lipid profiles, and lower cortisol responses under stress (see, e.g., Carver and Scheier, 2014). Data confirm how dispositional optimism can affect distress also in its biological aspects, keeping the immune system free from dysregulation and reactive to viral or bacterial infections.

Therefore, for example, the media should place greater emphasis on those who have recovered rather than new cases of infection and death, but even the World Health Organization website (World Health Organization [WHO], 2020) also reports data on confirmed cases, deaths and affected nations. Indeed, it is necessary to keep in mind that health and authority experts (virologists, immunologist, and epidemiologists) together with journalists are creators of the information conveyed through the media. These authors of information should choose and product good and positive information that could be understood and “reached” by general population. Information can be collected and transferred, for example, from COCHRANE a global independent network of researchers, professionals, patients, carers, and people interested in health (Cochrane, 2020).

Finally, as in every moment of crisis, we should not forget the etymology of the word: crisis is an agricultural term that derives from the Greek verb krino, to separate, to group – in a broader sense, to discern, to judge, to evaluate. The verb was used in reference to threshing, which involves separating the grain from the straw and chaff, that is, the envelope covering the grain of wheat. This gave both the first meaning of “to separate” and the metaphorical meaning of “to choose.” It is therefore possible to grasp its positive nuance, since a crisis can be a period of reflection, evaluation, discernment, and become a prerequisite for a rebirth, for a next flourishing.

Therefore, from this umpteenth crisis, let us try to take the opportunity for growth, beyond the leveling and the habituation, the waiting for the next black swan, the next fear, the next distress, the next anxiety. Because we are all dependent on each other and we are all responsible for each other. Let us think of ourselves as we really are: *waves of the same sea, leaves of the same tree, flowers of the same garden.*

## Author Contributions

The author confirms being the sole contributor of this work and has approved it for publication.

## Conflict of Interest

The author declares that the research was conducted in the absence of any commercial or financial relationships that could be construed as a potential conflict of interest.
